# Analysis of patient’s X-ray exposure in hepatic chemosaturation procedures: a single center experience

**DOI:** 10.1186/s12880-022-00887-2

**Published:** 2022-09-13

**Authors:** Sebastian Ebel, Martin Reinhardt, Anne Bettina Beeskow, Felix Teske, Manuel Florian Struck, Rhea Veelken, Florian van Boemmel, Thomas Berg, Michael Moche, Matthias Gutberlet, Holger Gößmann, Timm Denecke

**Affiliations:** 1 Department of Diagnostic and Interventional Radiology, University Hospital Leipzig, Liebigstr. 20, 04103 Leipzig, Germany; 2 Department of Anesthesiology and Intensive Care Medicine, University Hospital Leipzig, Leipzig, Germany; 3 Section of Hepatology, University Hospital Leipzig, Leipzig, Germany; 4 Department of Interventional Radiology, Park Hospital, Leipzig, Germany; 5 Department of Diagnostic and Interventional Radiology, Heart Center Leipzig, Leipzig, Germany

**Keywords:** Percutaneous hepatic perfusion, Liver metastasis, Melanoma, Regional therapy, Inteventional radiology, Chemosaturation

## Abstract

**Background:**

Hepatic chemosaturation is a technique in which a high dose of the chemotherapeutic agent melphalan is administered directly into the liver while limiting systemic side effects. We reviewed our institutional experience regarding patient’s X-ray exposure caused by the procedure.

**Methods:**

Fifty-five procedures, performed between 2016 and 2020 in 18 patients by three interventional radiologists (radiologist), were analyzed regarding the patient’s exposure to radiation. Dose-area-product (DAP) and fluoroscopy time (FT) were correlated with the experience of the radiologist and whether the preprocedural evaluation (CS-EVA) and the procedure were performed by the same radiologist. Additionally, the impact of previous liver surgery on DAP/FT was analyzed.

**Results:**

Experienced radiologist require less DAP/FT (50 ± 18 Gy*cm^2^/13.2 ± 3.84 min vs. 69 ± 20 Gy*cm^2^/15.77 ± 7.82 min; *p* < 0.001). Chemosaturations performed by the same radiologist who performed CS-EVA required less DAP/FT (41 ± 12 Gy*cm^2^/11.46 ± 4.41 min vs. 62 ± 11 Gy*cm^2^/15.55 ± 7.91 min; *p* < 0.001). Chemosaturations in patients with prior liver surgery with involvement of the inferior cava vein required significantly higher DAP/FT (153 ± 27 Gy*cm^2^/25.43 ± 4.57 min vs. 56 ± 25 Gy*cm^2^/14.44 ± 7.55 min; *p* < 0.001).

**Conclusion:**

There is a significant learning curve regarding the procedure of hepatic chemosaturation. Due to dose reduction the evaluation and chemosaturation therapy should be performed by the same radiologist. Procedures in patients with previous liver surgery require higher DAP/FT.

## Introduction

Chemosaturation with percutaneous hepatic perfusion (CS-PHP) is a minimally invasive technique for controlling metastatic liver disease. The chemotherapeutic agent melphalan is administered directly into the hepatic arteries, the hepatic venous blood is then filtered by a special extracorporal filtration system and reintroduced via the jugular vein. Therefore, high doses of melphalan can be administered while minimizing systemic side effects [1, 2]. In the past years the feasibility and repeatability of CS-PHP in the setting of unresectable hepatic metastasis has been shown in numerous studies [2–9]. Especially in unresectable liver metastases of uveal melanomas CS-PHP has proven to be an effective and save therapeutic option [10].

In preparation of the actual CS-PHP a preprocedural angiography (CS-EVA) is performed routinely to evaluate the hepatic vascular anatomy, blood flow, and to exclude hepato-mesenterial or hepato-gastric shunts, with the potential need for embolization.

The procedure of CS-PHP requires exact arterial catheter positioning under fluoroscopic guidance; therefore it is accompanied by radiation exposure of the patient, the interventional Team consisting of the interventional radiologist, the anesthetists, the perfusionist and the assisting staff. Moreover, the exposure could be higher than in other interventions, e.g. trans-arterial chemoembolization (TACE) or selective internal radiotherapy (SIRT) because CS-PHP requires two additional venous accesses. The level of training of the radiologist has a significant influence of fluoroscopy time (FT) during several interventional procedures [11–13]. Additionally, the length of the supra-hepatic inferior vena cava (ICV) and alteration of the hepatic or vascular anatomy (congenital or acquired, e.g. due to previous liver surgery) may also prolong the FT and lead to a higher dose area products (DAP measured in Gy*cm^2^). Thus, we hypothesized that the FT might be shorter when the CS-EVA and the procedure of CS-PHP itself were performed by the same radiologist, due to prior knowledge of the patient specific vascular anatomy.

The purpose of this study was to evaluate the influence of the level of experience of the radiologist, anatomic variants, and whether the CS-EVA and CS-PHP were performed by the same radiologist, on FT and DAP during CS-PHP.

## Material and methods

### Study design

In this retrospective, single center study, datasets of 55 CS-PHP procedures, performed between 10/2016 and 10/2020 in 18 consecutive patients, were analyzed. All procedures were performed by three radiologist with experience in > 200 intraarterial procedures each.

Institutional ethics committee approval was obtained. Written and written informed consent was obtained from all patients. All methods were carried out in accordance with relevant guidelines and regulations.

All patients had undergone multiphasic CT and MRI of the abdomen prior to CS-PHP. All interventions were performed on a flat panel system (Azurion Clarity IQ, Philips Healthcare, Best, The Netherlands). The DAP meter of this system is calibrated to national standards in accordance with DIN 6868-150:2013-06.

**Fig. 1:**
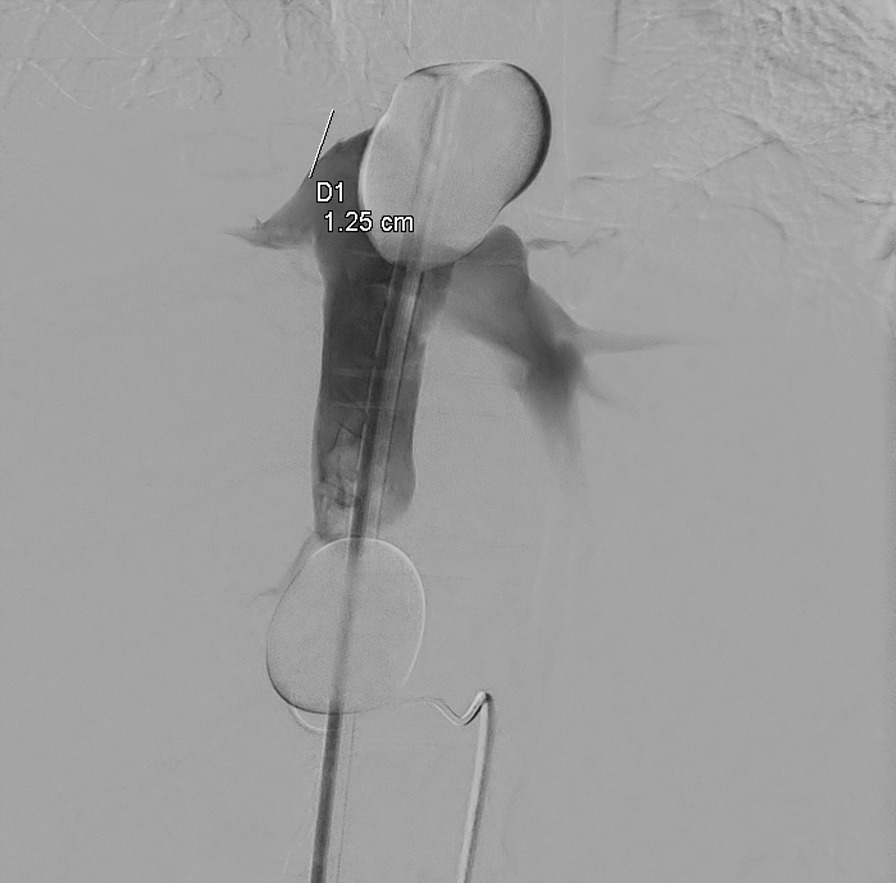
Measurement of the length of the supra-hepatic inferior cava vein performed on an angiogram to check for proper sealing of the double balloon catheter

All datasets were analyzed in terms of anatomical variances regarding the liver vasculature, i.e. an anomalous origin of the left or right hepatic artery. Additionally, we measured the length of the supra-hepatic ICV on the angiograms (Fig. 1). The ICV was defined as the region between the orifice of the most superior hepatic vein and the bottom of the right atrium. Furthermore, we analyzed if the patients underwent liver surgery prior to CS-PHP, and we analyzed whether the CS-EVA and CS-PHP were performed by the same radiologist.

All procedures were analyzed regarding FT and DAP and the number of digital acquisitions, provided by the software of the fluoroscopy unit (dose report), which was saved to our radiology information system (Syngo.plaza VB30C_HF4, Siemens, Erlangen, Germany).

### Procedure

CS-PHP was performed under general anesthesia and with systemic anticoagulation. The right common femoral artery (CFA) was accessed using a 4F angiography sheath, the right internal jugular vein (IJV) with a sheath and the right common femoral vein (CFV) using a 18F vascular sheath under ultrasonic guidance. A 4F catheter was placed in the common hepatic artery, then a micro-catheter was placed into the intended vessel for the administration of melphalan, based on the CS-EVA. A 16F double-balloon catheter (﻿Delcath Systems Inc., New York, NY) was positioned with its tip in the right atrium. This catheter has perforations between the two balloons for aspiration of the hepatic venous blood. The venous catheter was then connected to an extracorporeal filtration system. After all lines were in place, systemic anticoagulation (Heparin 300 IU / Kg) was applied until an activated clotting time (ACT) of > 450 s was maintained. Once the intended ACT was reached, the cranial balloon of the double balloon catheter was inflated inside the right atrium and retracted into the ICV, subsequently the caudal balloon was inflated inside the infrahepatic ICV, right below the orifice of the hepatic veins to prevent a systemic hepatovenous drain. Correct positioning of the two balloons was confirmed by a venous angiogram. Melphalan was administered at a dose of 3 mg/kg ideal body weight (maximum 220 mg/treatment) into the hepatic artery. The hepatic venous blood was aspired through the double-balloon catheter, filtered extracorporeally and returned through the IJV. The filtration was performed during and 30 min after the administration of melphalan.

### Image acquisition

The fluoroscopic framerate was 7.5 frames per second, the framerate for digital acquisitions was 2 frames per second. X-ray tube potential was 74 kV. ln 2 cases digital acquisitions were performed with beam angles of 10° cranial (CRAN) / 25° left-anterior-oblique (LAO) and 12° CRAN/20° LAO. All other cases were performed with beam angles of 0° CRAN/0° LAO.

### Statistical analysis

All analyses were performed using MedCalc Statistical Software V15.11.4 (MedCalc Software, Ostend, Belgium). Normally distributed quantitative variables were expressed as mean values and standard deviations (SD). Analysis included student’s *t* test, the Shapiro–Wilk test and Spearman’s correlation. Power analysis showed an overall Power of the study of 83%. A *p* value < 0.05 was considered statistically significant.

## Results

### Impact of the experience of the radiologist

**Fig. 2:**
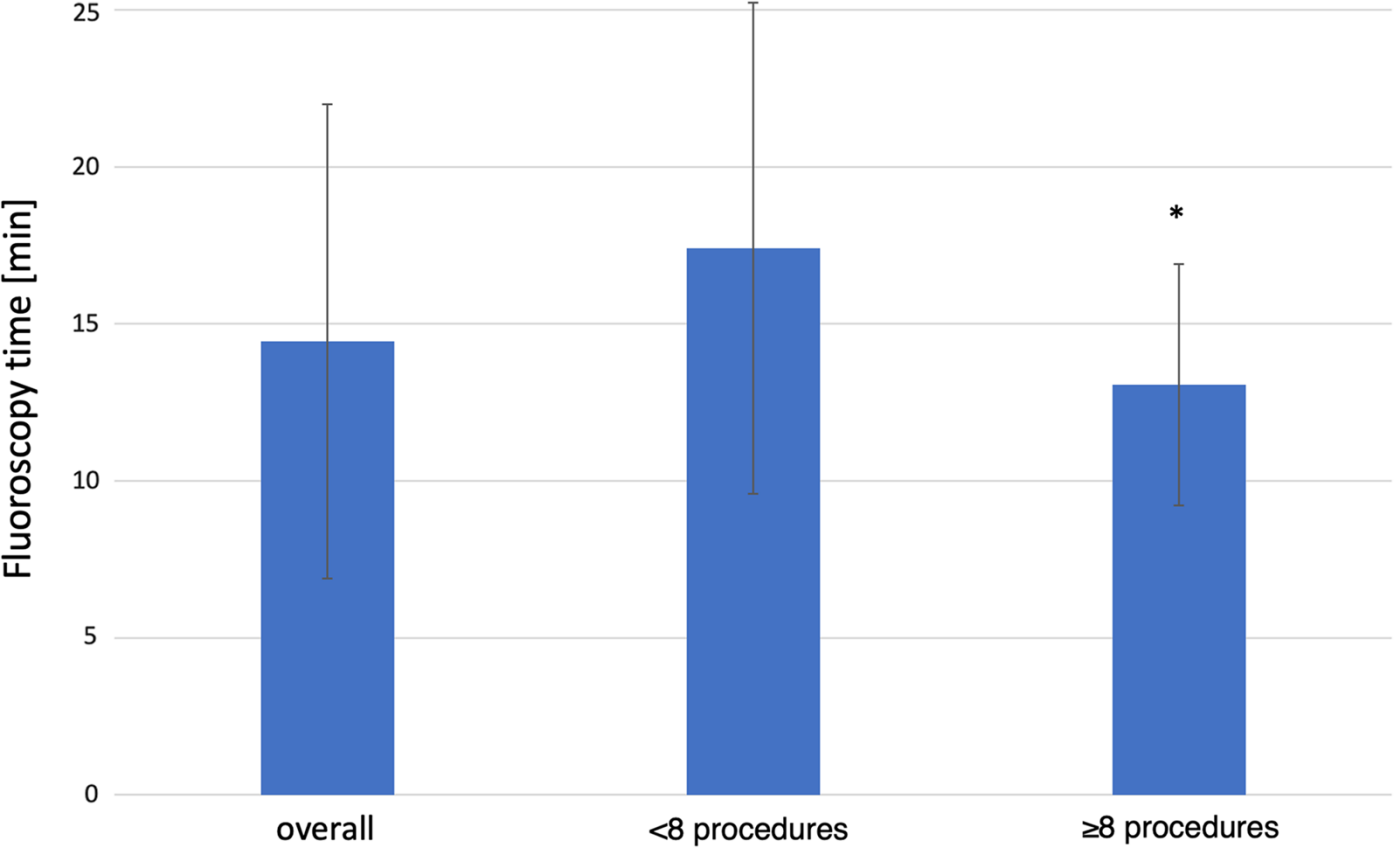
Box-plot comparison of the mean fluoroscopy time (min) overall and according to the radiologist experience. Significant differences are marked by *

**Fig. 3:**
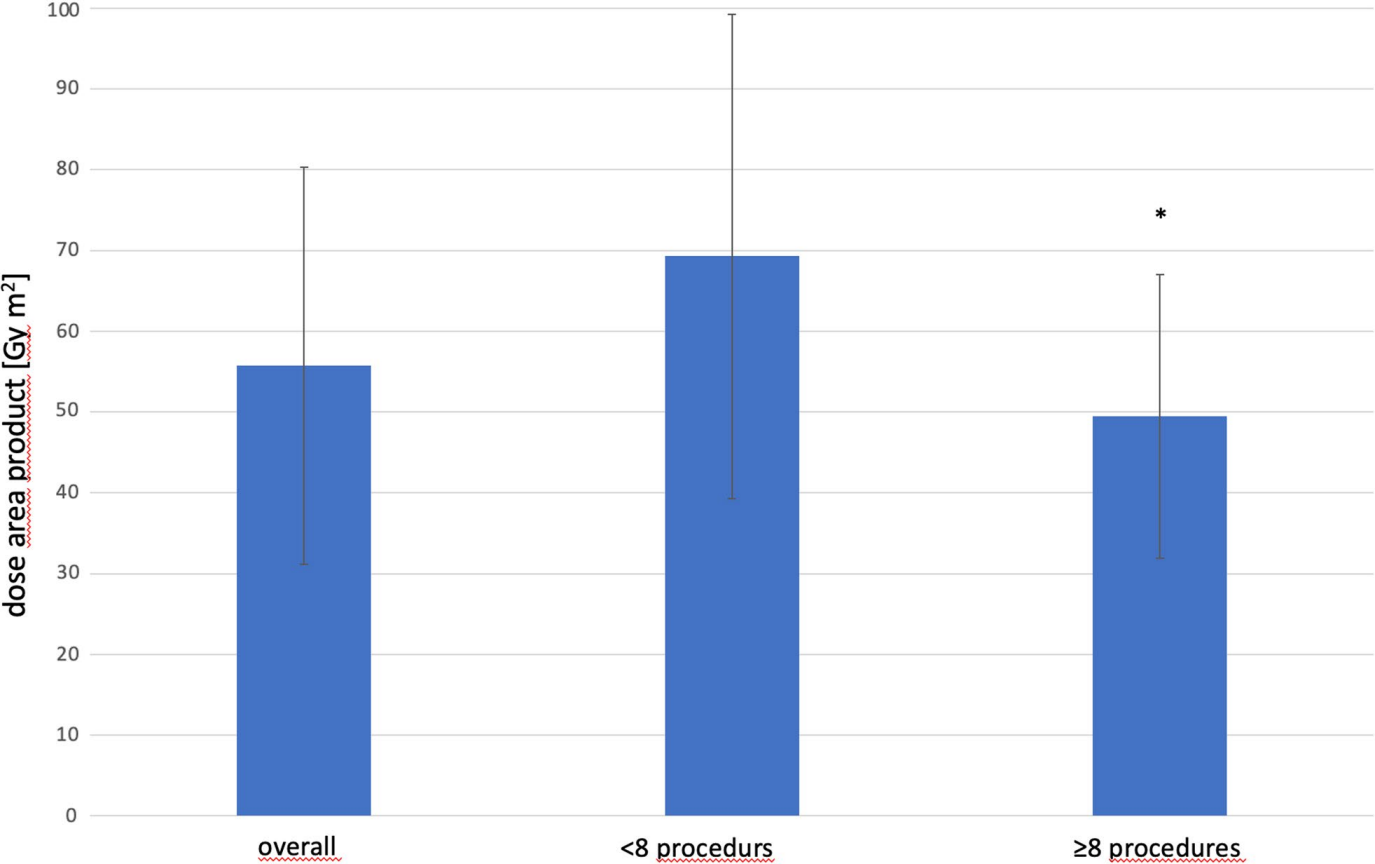
Box-plot comparison of the mean dose area product overall and according to the radiologist experience. Significant differences are marked by *

Three radiologists were investigated in this study. IR1 has performed 19 procedures, IR2 21 procedures and IR3 15 procedures of CS-PHP. Overall, the mean FT was 14.44 ± 7.55 min and DAP was 56 ± 25 Gy*cm^2^. Correlation analysis (number of procedures vs. FT/DAP) revealed a significant reduction of FT and DAP after the 7th procedure: Radiologists who performed less than 8 CS-PHP required more FT and DAP compared to more experienced radiologists (≥ 8 procedures): FT 13.2 ± 3.84 min vs. 15.77 ± 7.82 min; *p* < 0.01) and DAP (49 ± 18 Gy*cm^2^ vs. 69 ± 30 Gy*cm^2^; *p* < 0.01) with a mean DAP reduction of 29% and a median FT reduction of 17% (Figs. 2, 3). We found a strong negative correlation between DAP, FT and the number of procedures performed by the specific radiologist (R = − 0.71, *p* = 0.034). See Table 1. There were no significant correlation between the experience of the radiologist and the use of angled projections (R = 0.04, *p* = 0.31) and no link between experience and the number of digital acquisitions (R = 0.21, *p* = 0.43).

**Table 1:** Mean, minimum (min) and maximum (max) values of the dose area product (DAP) and the fluoroscopy time (FT) according to radiologist experience

	DAP [Gy*cm^2^]	FT [min]
< 8 performed procedures		
Min	43.2	12.4
Max	262.2	47.9
Mean	69.3	15.8
≥ 8 performed procedures		
Min	20	7.1
Max	110.1	19.1
Mean	49.5	13.2

### Impact of variabilities of the hepatic vasculature

We found anatomical variances regarding the hepatic arteries in 50% of the cases (n = 28): Origin of the left hepatic artery from the left gastric artery (n = 9; 16.1%), origin of the right hepatic artery from the superior mesenteric artery (SMA) (n = 13; 23.2%), separate origin of the segment-IV-artery (n = 6; 10.7%). We found no significant impact of arterial anatomical variances on FT and DAP (*p* > 0.05). In our cohort we found no anatomical anomalies regarding the hepatic veins.

The mean length of the ICV was 12.18 ± 3.57 mm. In our study we found no significant impact of the length of the VCI on FT or DAP (*p* > 0.05).

### Impact of prior surgery

In eight cases patients (14%) underwent liver surgery prior to CS-PHP: Segmentectomy (n = 3), lobectomy (n = 4), other resections (n = 1). Overall, we found no significant impact on FT and DAP. In three cases patch-repair of the ICV was performed during the surgery. In these cases, we found significant longer FT (25.43 ± 4.57 min; *p* < 0.01) and higher DAP (153 ± 27 Gy*cm^2^; *p* > 0.001) due to difficulties during the placement of the double balloon catheter. All three cases were performed by two of the investigated radiologists (procedure no. 11 and 15 (radiologist 1) and procedure no. 13 (radiologist 3).

### Impact of CS-EVA and CS-PHP performed by the same radiologist

**Fig. 4:**
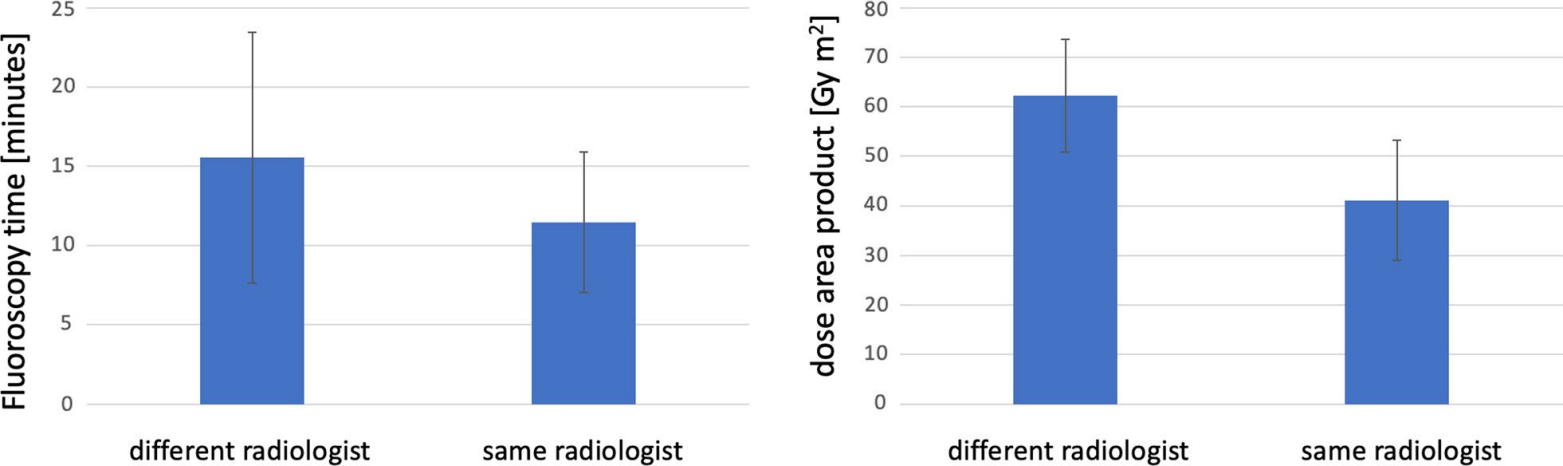
Box-plot comparison of the mean fluoroscopy time and dose area product with respect. If the preprocedural evaluation (CS-EVA) and the procedure were performed by different radiologist or the same radiologist. Significant differences are marked by *

When CS-Eva and CS-PHP were performed by different radiologists, mean FT and DAP was 15.55 ± 7.91 min and 62 ± 11.41 Gy*cm^2^. When the evaluation and CS-PHP were performed by the same radiologist mean FT was significant shorter (11.46 ± 4.41 min; *p* < 0.001) and DAP was significant lower (41 ± 12 Gy*cm^2^; *p* < 0.001) (Fig. 4).

## Discussion

In this study we analyzed the impact of different factors on the radiation dose during CS-PHP. In terms of patient, radiologist, and staff safety, FT and DAP should be kept as low as reasonably achievable (ALARA).

It is known that the interventionalists experience contributes to dose reduction: Jonczyk et al. cunducted a study in which they divided the participating interventionalists into a junior and a senior group (< vs. > 50 procedures) and they have shown that senior radiologists required significantly less FT (reduction of 42%) and DAP (reduction of during 16%) for the implantation of central venous port catheters than junior radiologists [13]. In our study we found a significant FT/DAP reduction after the 7th procedure with a mean DAP reduction of 29% and a median FT reduction of 17%. The higher dose reduction shown by Jonzcyk et al. might have been the result of higher complexity of the procedure of CS-PHP as compared to the implantation of central venous port catheters or because of the higher number of procedures as cut-off between junior and senior radiologists. Interestingly, Jonzcyk et al. found a stronger FT reduction compared to DAP reduction after 50 procedures, in contrast we found a stronger DAP reduction compared to FT reduction in the first 7 procedures: This could be interpreted as follows: during the first 8 procedures radiologists learn to work with higher collimation, lower image quality or and after the 7th procedure the learning curve changes and the interventionalists get faster. Contrarily, there was no correlation between the number of digital acquisitions and the radiologists experience. One possible explanation for this is, that the “challenges” of the procedure of CS-PHP are mostly due to difficult vessel catheterizations, which are performed under fluoroscopy and there are only few steps during the procedure that need actual digital acquisitions.

Anatomical variances of the arterial hepatic vasculature are common [14–16]. There are numerous studies about classification systems of variabilities of the hepatic arteries, highlighting the significance of knowledge about those variances for the daily practice of liver surgeons and interventional radiologists [17–19]. Our study reveals that these variabilities have no significant impact on the FT and DAP during CS-PHP. Because all patients underwent arterial phase scans (CT and MRI) for tumor staging and procedural planning as well, all radiologist knew about possible anatomical variabilities prior CS-PHP. Another possible explanation for this observation might be that all participating radiologist had the experience of more than 100 procedures each within the hepatic arteries, e.g. transarterial chemoembolization (TACE) and selective internal radiotherapy (SIRT). That means that all radiologist in this study were trained in the management of anatomic variations of the hepatic arteries. Our data is unable to explain whether “true” junior radiologists would have needed higher FT and DAP in the case of aberrant liver arteries.

The treatment of metastatic liver disease includes surgery, chemotherapy as well as interventional procedures. Often patients undergo a combination of multiple treatment approaches, e.g. tumor reductive surgery and additional CS-PHP [20]. In our study, eight cases (14%) patients underwent surgery prior CS-PHP of which in three cases patch-repair of the ICV was performed. The procedures in these three cases were performed by two of the investigated radiologist (case no. 11 and 15 of IR1 and case no. 13 of IR3. In all these cases the placement of the double balloon catheter was more difficult due to leakage at the upper balloon. Subsequently we found higher DAP and prolonged FT in all three cases.

To the best of our knowledge this is the first study reporting about the impact of previous liver surgery on the procedure of CS-PHP. Since CS-EVA only includes evaluation of the hepatic arteries (and not the hepatic veins) possible alterations with subsequent difficulties in positioning of the double balloon catheter will be noticed only during CS-PHP. Therefore, we recommend that when performing CS-PHP in patients who had undergone prior liver surgery, the thorough analysis of preprocedural CT and MR scans are mandatory. Additionally, more sophisticated techniques for detection of leakage of the double balloon catheters can be used, e.g. 2D-perfusion angiography as introduced by Dewald et al. [21].

Besides physical examination and laboratory tests, the preprocedural assessment prior to CS-PHP includes the performance of a visceral arteriography (CS-EVA) to elucidate the individial blood flow and to rule out hepato-mesenterial or hepato-gastric shunts. In our study, we found shorter FT and lower DAP during CS-PHP if the actual therapy and CS-EVA were performed by the same radiologist. A possible explanation for this result is that during the CS-EVA the radiologist becomes familiar with patient specific procedural and anatomical details, e.g. how the different catheters or guidewires work in the specific patient.

Limitations of this study include its retrospective design and the rather small number of included patients. Further studies should include patient dosimetry to calculate the patient’s effective dose. Because previous studies reporting on radiation exposure during CS-PHP are not available, we could not compare further studies should include a multicenter comparison. Additionally, the investigated radiologist were no “true beginners”; as mentioned above, they were trained in more than 100 transarterial hepatic procedures each (e.g. TACE and SIRT), but since the positioning of the venous doubleballoon catheter is a unique part of CS-PHP, which the investigated radiologist haven’t performed before, they can be considered as “true beginners” in this instance.

## Conclusion

In conclusion, there is a considerable learning curve regarding the procedure of CS-PHP which is associated with a dose reduction of radiation exposure. Thus, the angiographic evaluation for chemosaturation and the therapy itself should be performed by the same radiologist. Furthermore, previous liver surgery prior CS-PHP with involvement of the ICV might be a risk factor for higher DAP and longer FT.

## Data Availability

The datasets used and analysed during the current study are available from the corresponding author on reasonable request.

## Abbreviations

Radiologist: Interventional radiologist
DAP: Dose-area-product
FT: Flouroscopy time
CS-EVA: Chemosaturation evaluation
CS-PHP: Chemosaturation with percutaneous hepatic perfusion
TACE: Transarterial chemoembolization
SIRT: Selective internal radiotherapy
ICV: Inferior cava vein
CFA: Common femoral artery
IJV: Internal jugular vein
CFV: Common femoral vein
ACT: Activated clotting time
SMA: Superior mesenteric artery
ALARA: As low as reasonably achievable
CT: Computed tomography
MRI: Magnetic resonance imaging
